# Pretreatment albumin/fibrinogen ratio as a promising predictor for the survival of advanced non small-cell lung cancer patients undergoing first-line platinum-based chemotherapy

**DOI:** 10.1186/s12885-019-5490-y

**Published:** 2019-03-29

**Authors:** Jun Ying, Danfei Zhou, Tongjie Gu, Jianda Huang, Haijian Liu

**Affiliations:** Department of Respiratory, Hwa Mei Hospital, University of Chinese Academy of Sciences, NO. 41, Xibei Street, Ningbo, 315000 Zhejiang Province China

**Keywords:** Non small-cell lung cancers, Chemotherapy, Prognosis, Albumin-to-fibrinogen ratio

## Abstract

**Background:**

This study aimed to identify potential predictive factors for the survival of advanced non small-cell lung cancer (NSCLC) patients undergoing first-line platinum-based chemotherapy.

**Methods:**

A total of 270 advanced NSCLC patients who underwent first-line platinum-based chemotherapy from June, 2011 to June, 2015 were enrolled. A receiver operating characteristic (ROC) curve analysis was used to evaluate the predictive value of the albumin-to-fibrinogen ratio (AFR) for overall survival (OS). The predictive factors for survival were evaluated by univariate and multivariate analyses via the Cox proportional hazards regression model. The OS and progression free survival (PFS) results were determined via the Kaplan–Meier method using the log-rank analysis.

**Results:**

Based on the results of the ROC curve analysis, 8.02 was accepted as the cut-off AFR value for OS. The metastasis stage (M0 vs M1a/b, HR: 1.73, 95% CI: 1.15–2.59, *P* = 0.020) and AFR (≤8.02 vs > 8.02, HR: 1.80, 95% CI: 1.09–2.78, *P* = 0.025) were two independent risk factors for PFS by multivariate Cox regression analysis. The AFR (≤8.02 vs > 8.02, HR: 1.79, 95% CI: 1.11–2.59, *P* = 0.029) was a significant predictive factor for OS in advanced NSCLC patients. The PFS (*P* = 0.008) and OS (*P* = 0.003) in the high AFR group were significantly improved compared with those in the low AFR group via the Kaplan–Meier method using the log-rank analysis.

**Conclusions:**

The AFR could be a potential effective predictive factor for the survival in advanced NSCLC patients undergoing first-line platinum-based chemotherapy.

## Background

Lung cancer (LC) is consistently the leading cause of the cancer-related deaths worldwide [1]. Non small-cell lung cancer (NSCLC), the most common type of LC, compromises for nearly 85% of all LC cases [2]. For early-stage NSCLC, surgery has been widely accepted as the main therapeutic strategy; however, high postoperative recurrence and metastasis rates are commonly observed [3]. Most NSCLC patients are diagnosed at a relatively late stage (stage III or IV), which may preclude surgical resections [4]. Platinum-based chemotherapy, which consists of third-generation chemotherapy and platinum, is widely accepted as a standard treatment for advanced NSCLC [5]. In the past decades, new available therapeutic options for advanced NSCLC have been proposed and developed quickly. Recently, immunotherapy has been increasingly applied as clinical treatments for LC patients [6]. For example, immune checkpoint inhibitors (ICIs), such as anti-programmed death-1 (PD-1) or programmed death-ligand 1 (PD-L1) have been proven proved to be clinically effective across a broad range of solid tumors, including NSCLC [7]. Targeted therapy, such as anaplastic lymphoma kinase (ALK) inhibitors and tyrosine kinase inhibitors (TKIs), has also attracted much attention due to its improvements in tolerability and efficacy [8]. Other researchers have also suggested that the combination treatment strategies may maximize the treatment efficacy for NSCLC [7].

Due to individual differences in chemotherapy efficacy and the relatively high recurrence rate, an early prediction for chemotherapy response and clinical outcomes is urgently needed [9]. Investigating valid predictive biomarkers can aid in timely, effective intervention and better clinical efficacy. A minimally invasive predictor can serve as a tool for treatment monitoring and provide a new view on future guidance. Furthermore, it is also helpful to explore new and more effective therapeutic targets for advanced NSCLC. Various clinicopathological prognostic factors have been identified by previous studies, however, the prognosis prediction of NSCLC still remains unsatisfactory [10]. In recent decades, many studies have illustrated that inflammation and immunology are two important characteristics that enable malignancies and they are both significantly associated with tumour initiation, distant metastasis and resistance to chemotherapy [11, 12]. Furthermore, significantly different expressions of immune cells and inflammatory proteins such as albumin (Alb) and fibrinogen (Fib) are widely observed in LC patients in comparison with healthy controls [13]. Alb has been widely used as an effective tool for evaluating nutritional and inflammatory status. In addition, recent evidence with various malignancies has also revealed a close relation between coagulation and tumor progression and metastasis [14, 15]. Fib is an essential protein in the coagulation cascade and it has been reported by some studies to be associated with tumor development [16]. However, the correlation between the Alb-to-Fib ratio (AFR) and the prognosis of NSCLC with patients undergoing first-line platinum-based chemotherapy remains unknown until now. Hence, this study aimed to investigate whether the AFR could serve as an effective predictor for the survival of NSCLC patients.

## Methods

### Patients

This retrospective study was approved by the Medical Institutional Ethics Committee of Hwa Mei Hospital and Zhejiang province. Eligible patients admitted to the Department of Respiratory of Hwa Mei Hospital, University Of Chinese Academy Of Sciences from June, 2011 to June, 2015 were enrolled. The inclusion criteria were as follows: [1] newly diagnosed NSCLC patients with histopathological or cytological evidence; [2] advanced NSCLC patients in stage III_B_ and IV (including stage III_A_, which is not suitable for surgery) according to the tumor–nodes–metastasis (TNM) criteria (7th edition, AJCC criteria 2009) and [3] patients who underwent at least two cycles of first-lined platinum-based chemotherapy. The exclusion criteria were as follows: [1] patients who were pregnancy, or had brain metastases or concomitant malignancies; [2] patients with abnormal liver function, autoimmune or hematological diseases; [3] patients who had previous radiation therapy or surgery and [4] patients who did not have complete clinical data or who were lost to follow-up. Considering the impact of different treatment strategies on prognosis, the patients with radical-intent radiotherapy, surgery or who were not treated with platinum-based chemotherapy were also excluded.

### Treatment and follow up

All enrolled NSCLC patients were treated intravenously with first-line platinum-based chemotherapy, which consisted of platinum and third-generation chemotherapy agents (pemetrexed, docetaxel, and gemcitabine). The same regimen was repeated for an interval of 21 days for at least two cycles. Then, the therapy response of all patients was evaluated by the same independent radiologists and clinical physicians according to the revised Response Evaluation Criteria in Solid Tumors (RECIST) guideline (version 1.1) [17]. The invited radiologists were blinded to this study and they reviewed all scans of the enrolled patients to evaluate the therapy response. The follow-up duration was from the time chemotherapy was initiated to October 31, 2017.

### Prognosis evaluation

The therapeutic response was classified as complete response (CR), partial response (PR), stable disease (SD), and progressive disease (PD). Progression free survival (PFS) was calculated from the initiation of chemotherapy to disease progression, death or the set deadline of the study. The overall survival (OS) was defined as the period from the initiation of chemotherapy to death or the set deadline of the study. (Oct 31, 2017).

### Data collection

The clinical demographics (including age, gender, etc.), history of smoking, alcohol consumption habits, comorbidities (diabetes, hypertension), pathological characteristics (tumor size, lymph node, metastasis stage, etc.), gene characteristics, chemotherapy regimens, clinical response and laboratory test results were recorded for all NSCLC patients. Pretreatment fasting peripheral blood samples were collected at 7:00 am 1 day prior to chemotherapy. The obtained blood samples were processed within 48 h to detect biomarkers including blood cells, Alb, Fib, and C-reactive protein (CRP) levels. The AFR was calculated by dividing the Alb level by the Fib level.

### Statistical analysis

All the statistical analyses were performed using GraphPad Prism 5.0 (GraphPad Inc., San Diego, CA, USA) and SPSS 19.0 (SPSS Inc., Chicago, IL, USA). Data are summarized as the number and proportion, or mean ± standard deviation (SD). A receiver operating characteristic (ROC) curve analysis was used to evaluate the predictive value of AFR for OS, with respect to the cutoff value, sensitivity and specificity. Chi-squared tests, Student’s t tests or Mann–Whitney U tests were performed to compare baseline clinical characteristics as appropriate. The predictive factors for survival were evaluated by univariate and multivariate analyses via the Cox proportional hazards regression model. OS and PFS results were determined via the Kaplan–Meier method using the log-rank analysis. *P* < 0.05 was considered to be statistically significant.

## Results

### Patient characteristics

Based on the inclusion criteria, 297 NSCLC patients who underwent at least two cycles of first-line platinum-based chemotherapy were enrolled. Finally 270 were included into the analysis and the following 27 patients were excluded according to the exclusion criteria, 3 with concomitant malignancies, 4 with abnormal liver function, 3 with hematological diseases, 10 with incomplete data, and 7 lost to follow-up. The mean age of all enrolled patients was 59.4 years, and 173 (64.1%) patients were male. Seventy-eight (28.9%) patients were in stage III and 192 (71.1%) in stage IV, respectively.

### AFR and clinical characteristics

**Fig. 1:**
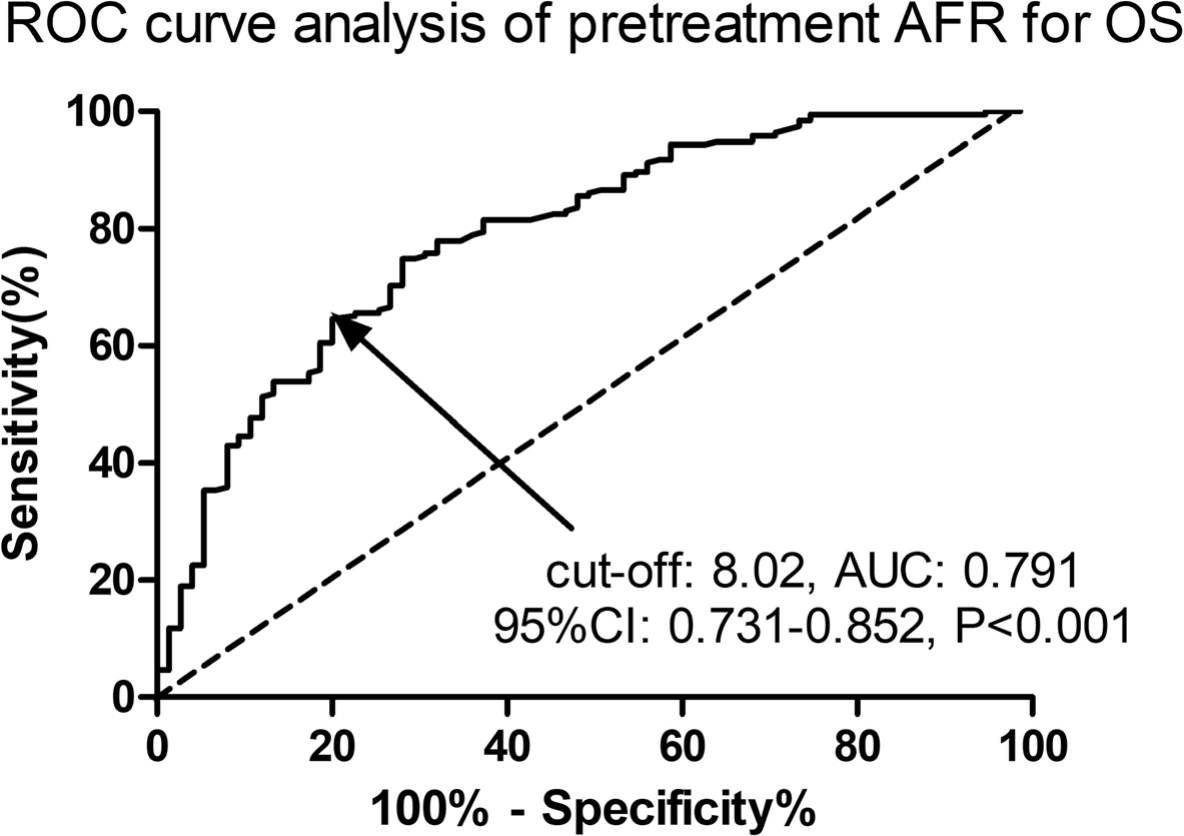
The ROC curve analysis of the AFR for OS. The pretreatment AFR was a potential predictor for OS with an AUC of 0.791, 95%CI of 0.731–0.852, a cut-off value of 8.02, a sensitivity of 65.64% and a specificity of 77.33% (*P* < 0.001). AFR, albumin-to-fibrinogen ratio; OS; overall survival; ROC, receiver operating characteristic; AUC, the area under the curve; CI, confidence interval

Based on the results of the ROC curve analysis, 8.02 was accepted as the cut-off AFR value for OS, with an area under the curve (AUC) of 0.791, a sensitivity of 65.64% and a specificity of 77.33%, respectively (Fig. 1). A total of 151 (55.9%) patients were categorized into the low AFR group (pretreatment AFR ≤8.02) and 119 (44.1%) in the high AFR group (pretreatment AFR > 8.02). The demographic and clinical characteristics associated with the AFR are presented in Table 1. The patients in the high and low AFR groups did not differ significantly from each other in age, gender, history of smoking, alcohol consumption habits, comorbidity morbidities, Eastern Cooperative Oncology Group performance status (ECOG PS), tumor location, tumor size, lymph node, metastasis stage, tumor–nodes–metastasis (TNM) stage, gene mutation status of EGFR,EML4-ALK and K-ras, histology or chemotherapy regimens (*P* > 0.05). Patients in the low AFR group were significantly associated with a higher prevalence of poorly differentiated tumors than those in the high AFR group (*P* = 0.039). Moreover, patients with an AFR value ≤8.02 had a worse clinical response to chemotherapy than patients with an AFR > 8.02 (*P* = 0.015). A significant difference of pretreatment CRP levels was also observed between the high and low AFR groups (*P* = 0.018).

**Table 1:** Demographic and clinical characteristics of NSCLC patients associated with AFR

Parameters	AFR ≤ 8.02	AFR > 8.02	*p* value
No. of patients	151	119	–
Age (years)	60.1 ± 10.7	58.5 ± 10.8	0.226
Gender (*n*, %)			0.180
Male	102(67.5)	71(59.7)	–
Female	49(32.5)	48(40.3)	–
Current smokers (*n*, %)	81(53.6)	52(43.7)	0.105
Alcohol (*n*, %)	34(22.5)	21(17.6)	0.308
Diabetes (*n*, %)	25(16.6)	13(10.9)	0.186
Hypertension (*n*, %)	32(21.2)	20(16.8)	0.364
ECOG PS (*n*, %)			0.641
0	100(66.2)	82(68.9)	–
≥ 1	51(33.8)	37(31.1)	–
Location of primary tumor (*n*, %)			0.586
Left	71(47.0)	52(43.7)	–
Right	80(53.0)	67(56.3)	–
Tumor size (*n*, %)			0.391
T1/T2	72(47.7)	63(52.9)	–
T3/T4	79(52.3)	56(47.1)	–
Lymph node (*n*, %)			0.089
N0	20(13.2)	25(21.0)	–
N1–3	131(86.8)	94(79.0)	–
Metastasis stage (*n*, %)			0.709
M0	45(29.8)	33(27.7)	–
M1a/M1b	106(70.2)	86(72.3)	–
TNM stage (*n*, %)			0.709
III	45(29.8)	33(27.7)	–
IV	106(70.2)	86(72.3)	–
Differentiation (*n*, %)			0.039*
Well/moderate	36(23.8)	42(35.3)	–
Poor	115(76.2)	77(64.7)	–
Histology (*n*, %)			0.448
SCC or others	104(68.9)	87(73.1)	–
AC	47(31.1)	32(26.9)	–
Chemotherapy (*n*, %)			0.085
DP	90(59.6)	83(69.7)	–
AP/GP	61(40.4)	36(30.3)	–
EGFR status			0.95
Wide-type	77(51.0)	63(52.9)	–
Mutation	43(28.5)	33(27.7)	–
Unknown	31(20.5)	23(19.3)	–
EML4-ALK status			0.93
Wide-type	102(67.5)	83(69.7)	–
Mutation	18(11.9)	13(10.9)	–
Unknown	31(20.5)	23(19.3)	–
K-ras status			0.77
Wide-type	93(61.6)	78(65.5)	–
Mutation	27(17.9)	18(15.1)	–
Unknown	31(20.5)	23(19.3)	–
Clinical response (*n*, %)			0.015*
CR/PR/SD	99(65.6)	94(79.0)	–
PD	52(34.4)	25(21.0)	–
Laboratory tests (*n*, %)			
Hemoglobin (g/L)	104.1 ± 13.3	106.3 ± 14.1	0.190
Platelet (10^9^/L)	177.4 ± 62.3	181.5 ± 75.8	0.626
WBC(10^9^/L)	7.2 ± 2.4	7.4 ± 2.6	0.513
CRP (ng/L)	8.4 ± 11.3	5.3 ± 9.7	0.018*

*NSCLC* Non–small cell lung cancer, *AFR* Albumin-to-fibrinogen ratio, *ECOG PS* Eastern cooperative oncology group performance status, *TNM* Tumor–node–metastasis, *SCC* Squamous carcinoma, *AC* Adenocarcinoma, *DP* Docetaxel combined with platinum, *AP* Pemetrexed combined with platinum, *GP* Gemcitabine combined with platinum, *CR* Complete response, *PR* Partial response, *SD* Stable disease, *PD* Progressive disease, *WBC* White blood cell, *CRP* C-reactive protein. *P*-values were calculated by Student’s t test, Mann–Whitney U test or Chi-squared test. * *P* < 0.05

### Predictive factors for PFS and OS

The potential risk factors for PFS and OS were evaluated by univariate and multivariate analyses using Cox proportional hazards regression model. Only factors with a *P* value < 0.1 in the univariate analysis were enrolled into the multivariate analysis. As shown in Table 2, the metastasis stage (M0 vs M1a/b, HR: 1.73, 95% CI: 1.15–2.59, *P* = 0.020) and AFR (≤8.02 vs > 8.02, HR: 1.80, 95% CI: 1.09–2.78, *P* = 0.025) were two independent risk factors for PFS by multivariate Cox regression analysis. The resulting predictive factors for OS are presented in Table 3, and the AFR (≤8.02 vs > 8.02, HR: 1.79, 95% CI: 1.11–2.59, *P* = 0.029) was found to be a significant predictive factor for OS in advanced NSCLC patients.

**Table 2:** Risk factors for PFS by univariate and multiple Cox proportional hazards regression analysis

	Univariate Multivariate			
Parameters	HR(95% CI)	*p* value	HR(95% CI)	*p* value
Age (high vs low)	1.18(0.98–1.46)	0.089	1.14(0.91–1.41)	0.131
Gender (male vs female)	1.12(0.72–1.73)	0.523		
Current smokers (yes vs no)	1.33(0.82–2.05)	0.251		
Alcohol (yes vs no)	1.03(0.83–1.29)	0.754		
Diabetes (yes vs no)	1.21(0.94–1.52)	0.122		
Hypertension (yes vs no)	1.47(0.79–2.58)	0.132		
ECOG PS (0 vs ≥1)	0.86(0.71–1.07)	0.845		
Tumor size (T1/T2 vs T3/T4)	0.78(0.39–1.55)	0.461		
Lymph node (N0 vs N1–3)	1.92(0.89–3.78)	0.058	1.53(0.69–2.87)	0.205
Metastasis stage (M0 vs M1a/b)	1.79(1.15–2.64)	0.011*	1.73(1.15–2.59)	0.020*
Differentiation (Well/moderate vs poor)	1.74(1.03–2.88)	0.039*	1.60(0.89–2.73)	0.089
Histology (AC vs non-AC)	0.85(0.51–1.29)	0.407		
Chemotherapy (DP vs AP/GP)	1.52(0.74–3.05)	0.245		
CRP (high vs low)	0.86(0.71–1.07)	0.845		
AFR (≤8.02 vs > 8.02)	2.07(1.32–3.12)	0.009*	1.80(1.09–2.78)	0.025*

*NSCLC* Non–small cell lung cancer, *ECOG PS* Eastern cooperative oncology group performance status, *TNM* Tumor–node–metastasis, *SCC* Squamous carcinoma, *AC* Adenocarcinoma, *DP* Docetaxel combined with platinum, *AP* Pemetrexed combined with platinum, *GP* Gemcitabine combined with platinum, *CRP* C-reactive protein, *AFR* Albumin-to-fibrinogen ratio, *PFS* Progression free survival, *HR* Hazard ratio, *CI* Confidence interval. *P < 0.05

**Table 3:** Risk factors for OS by univariate and multiple Cox proportional hazards regression analysis

	Univariate Multivariate			
Parameters	HR(95%CI)	*p* value	HR(95%CI)	*p* value
Age (high vs low)	1.13(0.57–2.17)	0.587		
Gender (male vs female)	1.49(0.76–2.78)	0.224		
Current smokers (yes vs no)	1.11(0.60–1.97)	0.754		
Alcohol (yes vs no)	1.42(0.75–2.55)	0.215		
Diabetes (yes vs no)	1.78(0.61–4.45)	0.287		
Hypertension (yes vs no)	1.14(0.53–2.51)	0.678		
ECOG PS (0 vs ≥1)	0.88(0.45–1.61)	0.612		
Tumor size (T1/T2 vs T3/T4)	1.67(0.92–3.15)	0.089	1.88(0.92–3.58)	0.073
Lymph node (N0 vs N1–3)	1.92(0.87–3.98)	0.142		
Metastasis stage (M0 vs M1a/b)	2.06(1.07–3.88)	0.024*	1.73(0.90–2.92)	0.093
Differentiation (Well/moderate vs poor)	1.55(1.04–2.36)	0.041*	1.22(0.81–1.72)	0.312
Histology (AC vs non-AC)	1.24(0.70–2.14)	0.412		
Chemotherapy (DP vs AP/GP)	1.43(0.88–2.45)	0.126		
CRP (high vs low)	1.39(0.78–2.43)	0.244		
AFR (≤8.02 vs > 8.02)	1.93(1.28–2.98)	0.011*	1.79(1.11–2.59)	0.029*

*NSCLC* Non–small cell lung cancer, *ECOG PS* Eastern cooperative oncology group performance status, *TNM* Tumor–node–metastasis, *SCC* Squamous carcinoma, *AC* Adenocarcinoma, *DP* Docetaxel combined with platinum, *AP* Pemetrexed combined with platinum, *GP* Gemcitabine combined with platinum, *CRP* C-reactive protein, *AFR* Albumin-to-fibrinogen ratio, *OS* Overall survival, *HR* Hazard ratio, *CI* Confidence interval. *P < 0.05

### AFR and survival analysis

**Fig. 2:**
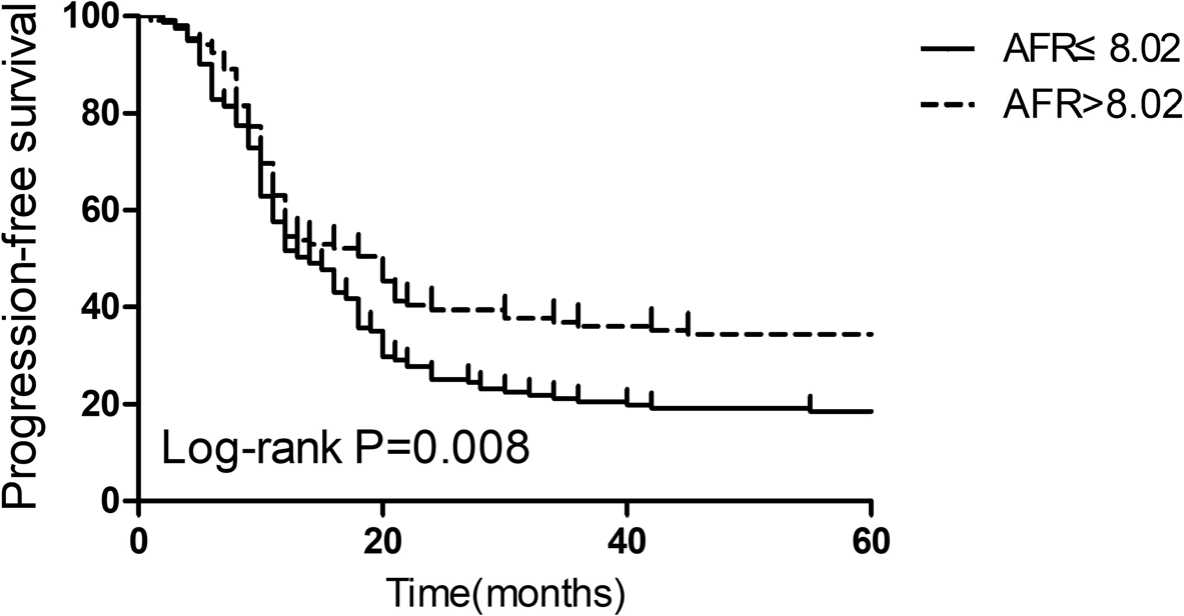
Kaplan-Meier curves of progression-free survival in advanced NSCLC patients stratified by the AFR. A low AFR was significantly associated with a worse progression-free survival than a high AFR (*P* = 0.008). AFR, albumin-to-fibrinogen ratio; NSCLC, non small-cell lung cancers

**Fig. 3:**
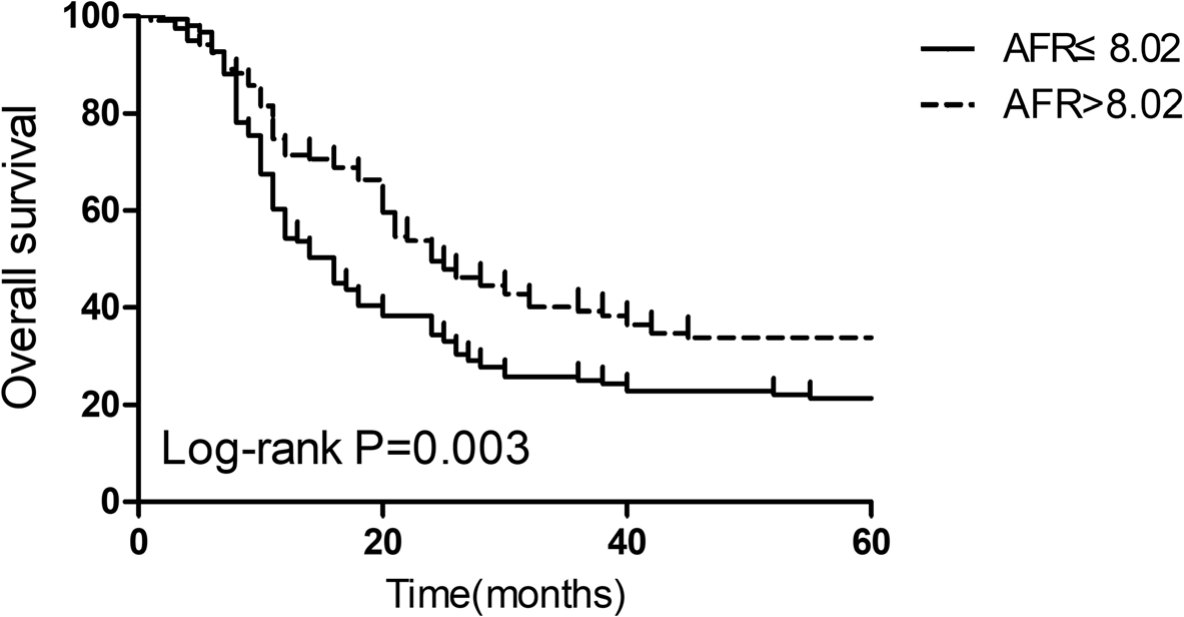
Kaplan-Meier curves of overall survival in advanced NSCLC patients stratified by AFR. A low AFR was significantly associated with a worse overall survival than a high AFR (*P* = 0.003). AFR, albumin-to-fibrinogen ratio; NSCLC, non small-cell lung cancer

To further explore the association between the AFR and survival in advanced NSCLC patients, the Kaplan-Meier curves of PFS and OS were calculated. As shown in Figs. 2 and 3, the PFS (*P* = 0.008) and OS (*P* = 0.003) were significantly improved in the high AFR group compared with the low AFR group by log-rank tests.

## Discussion

As demonstrated by recent studies, inflammation and nutritional status play key roles in tumor progression [18]. It is also well known that nutritional status and chronic inflammation are two main causes for NSCLC [19]. This current study focused on the potential predictive factors for prognosis in advanced NSCLC patients who underwent first-line platinum-based chemotherapy. Our results showed that the pretreatment AFR was an independent risk factor for both PFS and OS in advanced NSCLC patients. Patients with a lower pretreatment AFR had a worse clinical response to for chemotherapy, PFS and OS than patients with a high pretreatment AFR. In addition to the AFR, the present study also indicated that metastasis stage is a significant prognostic factor for PFS.

Albumin, a sensitive marker in nutritional status assessments, is an acute-phase reactant for systemic inflammation status and its synthesis is suppressed by inflammatory cytokines [20]. A systematic review of the epidemiological literature reveals the significant prognostic role of pretreatment serum albumin in cancer and baseline albumin levels that are recommended for risk stratification [21]. The close association between inflammation and tumors has attracted much attention. Inflammation in the tumor microenvironment plays important roles in the proliferation, survival, angiogenesis and metastasis of malignant cells [18]. Inflammation also accounts for the response alterations of the adaptive immune system to chemotherapeutic agents [18]. It has been demonstrated that malnutrition status is closely related to the progression of malignancy due to its association with high leukocytes levels, high risks of infection, and weak immune systems [21, 22].

Fib, an essential constituent of the coagulation system, plays an important role in platelet aggregation and blood coagulation. Furthermore, Fib is also widely accepted as a reliable biomarker that reflects systemic inflammation and Fib can promote the synthesis of pro-inflammatory cytokines [23]. High Fib expression induced by the excess production of inflammatory cytokines may be associated with tumor aggressiveness in NSCLC patients [24]. Plasma Fib contributes to the coagulation status and promotes the survival and adhesion of tumor cells, which results in LC metastasis [25]. Elevated Fib levels are reported to be tightly associated with high risk of colorectal, lung and breast cancer [26]. Preoperative serum Fib levels are suggested to be a candidate prognostic biomarker in operable NSCLCs [27].

Other studies have indicated the prognostic roles of plasma fibrinogen [28] and albumin [29] for survival in NSCLC patients. Our results showed that the AFR, which takes both albumin and fibrinogen into account, served as a strong predictor for prognosis. The AFR is an indicator of nutritional status, coagulation condition, and systemic inflammation. It has been indicated that the interactions between the inflammatory response and tumor cells promote tumor progression in the host environment [30]. Previous studies have reported that inflammatory cytokines are involved in the proliferation, invasion and metastasis of tumor cells in various cancers [15]. A recent study in advanced esophageal cancer patients reported that changes in AFR can serve as an independent risk factor for prognosis [31]. Moreover, in comparison with single Alb or Fib measurements, the AFR could amplify the sensitivity of nutritional and inflammatory status changes in NSCLC patients. Importantly, pretreatment AFR could clearly predict the clinical response to chemo-radiotherapy, which could partly explain its predictive role for survival. The AFR closely correlates with blood coagulation, nutritional and systemic inflammatory status, which may be possible explanations for the predictive role of AFR for survival in advanced NSCLC patients. In addition, some studies have indicated that chronic inflammation-associated biomarkers (such as elevated neutrophil count, CRP, etc.) can be potential predictors for nivolumab therapy in NSCLC [32]. The status of KRAS mutation has also been suggested as a prognostic biomarker for advanced NSCLC patients undergoing ICIs [33]. Some molecular predictive markers have been applied for NSCLC patients with platinum-based chemotherapy, such as excision repair cross-complementing 1 (ERCC1) [34], ribonucleotide reductase subunit M1 (RRM1) [35], class III β-tubulin (TUBB3) [36] and breast cancer 1 (BRCA1) expression [37]. Another circulating biomarker, the neutrophil-to-lymphocyte ratio (NLR) has been recommended as a potential predictor for outcomes in NSCLC patients treated with nivolumab [38]. Scilla et al. have also indicated that the baseline NLR is a novel prognostic factor in locally advanced NSCLC patients undergoing definitive chemoradiation with or without surgery [39]. However, whether these circulating biomarkers can serve as potential predictive/prognostic markers for NSCLC remains controversial and further validation is required for the clinical application for these markers.

We must admit that this study has some limitations. First, this is a signal-center retrospective study with a small sample size and the results require further validation in larger prospective data sets. Second, the observed sample cohort was quite specific and the lack of control group analyses (e.g. patients who were or were not receiving radiotherapy with platinum-based chemotherapy) was a great limitation for this study. Next, we will perform a study to investigate whether the AFR can serve as a prognostic factor for advanced NSCLC patients undergoing different treatment strategies (e.g. immunotherapy, targeted agents, combination therapy, etc.).

## Conclusions

In summary, our results indicated that the AFR could be a potential effective predictive factor for the survival in advanced NSCLC patients undergoing first-line platinum-based chemotherapy. However, more relevant studies with a larger sample sizes are warranted to validate our conclusions and explore the potential mechanisms.

## Abbreviations

AC: Adenocarcinoma
AFR: Albumin-to-fibrinogen ratio
Alb: Albumin
ALK: Anaplastic lymphoma kinase
AP: Pemetrexed combined with platinum
AUC: Area under the curve
CI: Confidence interval
CR: Complete response
CRP: C-reactive protein
DP: Docetaxel combined with platinum
ECOG PS: Eastern cooperative oncology group performance status
GP: Gemcitabine combined with platinum
HR: Hazard ratio
ICIs: Immune checkpoint inhibitors
LC: Lung cancer
NSCLC: Non–small cell lung cancer
OS: Overall survival
PD: Progressive disease
PD-1: Programmed death-1
PD-L1: Programmed death-ligand 1
PFS: Progression free survival
PR: Partial response
RECIST: Response Evaluation Criteria in Solid Tumors
ROC: Receiver operating characteristic
SCC: Squamous carcinoma
SD: Stable disease
TKIs: Tyrosine kinase inhibitors
TNM: Tumor–nodes–metastasis
WBC: White blood cell
